# Simultaneous Voltammetric Determination of Non-Steroidal Anti-Inflammatory Drugs (NSAIDs) Using a Modified Carbon Paste Electrode and Chemometrics

**DOI:** 10.3390/s23010421

**Published:** 2022-12-30

**Authors:** Guadalupe Yoselin Aguilar-Lira, Jesús Eduardo López-Barriguete, Prisciliano Hernandez, Giaan Arturo Álvarez-Romero, Juan Manuel Gutiérrez

**Affiliations:** 1 Laboratory of Analytical Chemistry, Academic Area of Chemistry, Institute of Basic Sciences and Engineering, Autonomous University of the State of Hidalgo, Pachuca 42076, Hidalgo, Mexico; 2 Bioelectronics Section, Department of Electrical Engineering, CINVESTAV-IPN, Mexico City 07360, Mexico; 3 Engineering and Energy Laboratory, Energy Area, Polytechnic University of Francisco I. Madero, Pachuca 42640, Hidalgo, Mexico

**Keywords:** non-steroidal anti-inflammatory drug, carbon paste electrode, differential pulse voltammetry, partial least square regression, artificial neural network, quantification

## Abstract

This work presents the simultaneous quantification of four non-steroidal anti-inflammatory drugs (NSAIDs), paracetamol, diclofenac, naproxen, and aspirin, in mixture solutions, by a laboratory-made working electrode based on carbon paste modified with multi-wall carbon nanotubes (MWCNT-CPE) and Differential Pulse Voltammetry (DPV). Preliminary electrochemical analysis was performed using cyclic voltammetry, and the sensor morphology was studied by scanning electronic microscopy and electrochemical impedance spectroscopy. The sample set ranging from 0.5 to 80 µmol L^−1^ was prepared using a complete factorial design (3^4^) and considering some interferent species such as ascorbic acid, glucose, and sodium dodecyl sulfate to build the response model and an external randomly subset of samples within the experimental domain. A data compression strategy based on discrete wavelet transform was applied to handle voltammograms’ complexity and high dimensionality. Afterward, Partial Least Square Regression (PLS) and Artificial Neural Networks (ANN) predicted the drug concentrations in the mixtures. PLS-adjusted models (*n* = 12) successfully predicted the concentration of paracetamol and diclofenac, achieving correlation values of R ≥ 0.9 (testing set). Meanwhile, the ANN model (four layers) obtained good prediction results, exhibiting R ≥ 0.968 for the four analyzed drugs (testing stage). Thus, an MWCNT-CPE electrode can be successfully used as a potential sensor for voltammetric determination and NSAID analysis.

## 1. Introduction

**Figure 1:**
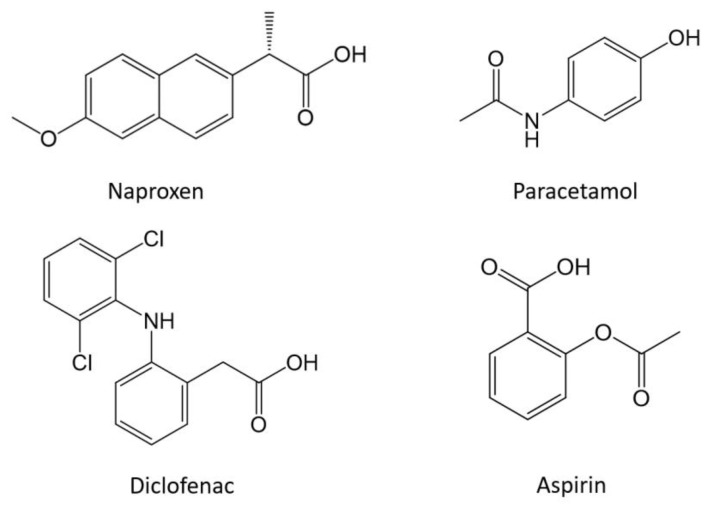
Chemical structure of four main NSAIDs.

Non-steroidal anti-inflammatory analgesics (NSAIDs) are important drugs worldwide due to their low cost and easy accessibility. Most of these drugs can be purchased without a prescription; they are widely used to relieve pain, reduce inflammation, and reduce high temperatures [1]. Standing out from this large group of NSAIDs are paracetamol, diclofenac, naproxen, and aspirin, which are the most frequently used. Figure 1 shows some examples of their chemical structure. These NSAIDs share the same pharmacological action, they block the enzyme cyclooxygenase and break down the prostaglandins produced by the cells of the body that increase inflammation, pain, and fever. Although NSAIDs are commonly used, they are not suitable for everyone and can sometimes cause adverse side effects if their use is constant, including peptic ulceration, digestive disorders, and temporary deafness. Recent studies mention that they may be related to heart attacks [2,3].

After use, NSAIDs are excreted in their native form or as metabolites, entering aquatic ecosystems in the concentration range of ng L^−1^–µg L^−1^. Although the concentrations of NSAIDs in surface waters are low, the high-activity biological properties of these molecules can confer potential toxicity to aquatic organisms converting them into emerging pollutants [4,5]. Therefore, the need for simple and fast simultaneous quantifications in the water of anti-inflammatory drugs represents a great challenge for water quality control, mainly when using electrochemical methods combined with sensors built with structurally different forms of carbon [6].

In 2020, approximately 30 million people used them daily worldwide, increasing their consumption [7,8]. Due to NSAIDs’ high demand and their impact on public health and ecosystems, many analytical methods have been proposed for their quantification. The most common method is high-performance liquid chromatography (HPLC) because it applies to many pharmaceutical compounds [9,10,11,12,13]. This method has some disadvantages, such as the need for sample preparation by chemical reaction or extraction. In addition to this, it requires considerable amounts of organic solvents for sample elution. Some cases include previous derivatization, time-consuming analysis, and a high cost associated with the use and maintenance of the equipment [14,15].

Considering these drawbacks, developing fast, reliable, and simultaneous determination procedures for these pharmaceutical samples remains a significant challenge in analytical chemistry. One approach to overcome the described shortcomings is the use of electrochemical sensors since most of these drugs are electroactive substances. Electrochemical sensors can provide advantages, such as being cheap and easy to use, having low detection limits, wide linear response ranges, good stability, reproducibility, direct detection of analyte, easy sample preparation, and the possibility of miniaturization [16].

In recent decades, electrochemical sensors coupled with voltammetric techniques such as Cyclic Voltammetry (CV) and Differential Pulse Voltammetry (DPV), were the most widely used because they present interesting characteristics for the determination of NSAIDs, dopamine, uric acid, ascorbic acid, among others, such as high sensitivity, high selectivity and accuracy, as well as low cost, and shorter analysis time compared to mass spectroscopy and HPLC [7,17,18].

The working electrode is very important in voltammetric methods because the redox reactions are carried out on it. In the literature, there are different types of electrodes, such as metallic electrodes, carbon paste electrodes (CPE), and glassy carbon electrodes [19,20]. CPE electrodes especially present multiple advantages because they have an electrochemical performance similar to pure metal electrodes responding to a wide voltage range. Further, their surface could be modified to improve selectivity and be renewed easily without the need for dangerous acids or bases for cleaning. They can be manufactured in any laboratory with the desired shape, even considering miniaturized dimensions, achieving high reproducibility [21].

Particularly, the modification of the CPE sensors has given rise to several innovations; among them, the most relevant modification is using carbon nanotubes (CNTs). Adding CNTs to formulate CPE sensors makes possible more stable and robust working electrodes. CNTs present high mechanical resistance and high electrical conductivity, such as copper, but with the ability to carry much higher currents. All these characteristics help to improve the electron transference in electrochemical reactions, making using modified paste electrodes with CNTs a very interesting option to solve different analytical problems [7,15,17,22,23].

Nevertheless, voltammetry presents a problem when analyzing more than one analyte simultaneously since the measured voltammograms are extraordinarily complex and generally contain a large volume of data [24,25,26,27].

Modeling voltammetric signals requires a chemometric approach to perform tasks focused on discriminating or quantifying analytes. Therefore, most voltammograms need a data reduction processing step before building an interpretation model. Techniques such as Principal Component Analysis (PCA), Discrete Fourier Transform (DFT), or Wavelet Transform (WT) are commonly used to enhance data, avoid redundancy, and facilitate data interpretation [28,29,30,31,32]. Finally, applying some multivariate methods or artificial intelligence techniques can model these improved voltammetric records. For quantitative applications, the most widespread approaches are Partial Least Squares Regression (PLS) and Artificial Neural Networks (ANNs) [33]. PLS is a technique that combines characteristics from multiple linear regression and PCA. It is helpful in the prediction of dependent variables from a large set of independent variables. It can analyze different sorts of data such as strongly correlated (collinear), noisy experimental, numerous X- variables, and several response variables (Y). Additionally, it can dissect incomplete variables from input and output matrix data. This method was originally introduced as an approximation model for linear regression [34,35]. It was originally used for economics and social science, but in recent years, it has been applied in different areas of chemical analysis [36,37]. Another way of interpretation is that the PLS technique is a model of similarities. Data measured on an array of similar samples, items, etc., can be processed to obtain approximations (PLS modeling). Then, fewer components need the model if the observations are more similar (samples, items, etc.) [38].

Conversely, ANN is a computational model that mimics how neurons work in the human brain. ANN is fascinating due to its adaptability to almost any mathematical transform. The models obtained can be linear or non-linear, which allows them to adapt to different types of sensors, making them useful for multivariable calibration and classification tasks. The most common ANN model is the Multilayer Perceptron (MLP), a supervised network that works with the backpropagation algorithm [39]. The error obtained by iteration is back-projected onto the network to minimize the error. The forward propagation network consists of the input layer, a hidden layer (one or more), and an output layer. The input layer units receive input signals and transfer the signal to the hidden layers via weighted weights. The output layer obtains these signals and provides the representative output. The input data mapping is carried out through the hidden layers of the MLP, and then the maximum representative signal is generated through the output layer [40].

For NSAID determination, most of the literature focuses on quantifying at least one drug in laboratory samples under controlled experimental conditions. Meanwhile, several works report different manufacturing strategies for sensors and biosensors to improve the detection limit by studying real-world samples. On the other hand, while some authors’ efforts have been placed on refining analytical methods to overcome limitations in detection, only a few papers reported the use of voltammetric methods coupled with chemometric approaches for the simultaneous quantification of NSAIDs [41].

The present work proposes a methodology based on a voltammetric sensor (made of carbon paste modified with carbon nanotubes) and the use of DPV due to its vast sensitivity, high selectivity, and accuracy, in addition to short analysis time, low cost, and no need of special sample pretreatment. Furthermore, CV is conducted to study the electrochemical profile of NSAIDs and the characterization of the working electrode. This technique is simple, fast, and brings sufficient information about different electroactive species of a sample. A morphological and impedance characterization by scanning electron microscopy (SEM) and electrochemical impedance spectroscopy (EIS) is developed to provide relevant details of the mixture (presence and transfer) on the surface of the modified electrode. Finally, a chemometric analysis is performed as a pre-processing data stage using Discrete Wavelet Transform (DWT) coupled with calibration models built by PLS and ANN to quantify paracetamol, diclofenac, naproxen, and aspirin simultaneously, showing a better selectivity and determination in the results of analysis and prediction.

## 2. Materials and Methods

### 2.1. Chemical and Reagents

All chemical reagents employed were of analytical grade and were acquired from Sigma-Aldrich (St. Louis, MO, USA). For building the sensor, multi-walled carbon nanotubes (MWCNT) (carbon base > 95%, O.D × L 6–9 nm × 5 µm) (CAS 308068-56-6), together with graphite powder (particle size < 20 μm) (CAS 7782-42-5) and mineral oil (CAS 8042-47-5), were used.

On the other hand, the solutions required were prepared using high-purity deionized water (18.2 MΩ cm) from Milli-Q (Millipore, Bedford, USA). In addition, NSAID standard solutions of Paracetamol (CAS 103-90-2), Diclofenac sodium (CAS 15307-79-6), Naproxen sodium (CAS 26159-34-2), and Aspirin (CAS 50-78-2), as well as Redox and non-Redox interferents L-Ascorbic acid (CAS 50-81-7), Lactose (CAS 63-42-3), D-(+)-Glucose (CAS 50-99-7), and Sodium dodecyl sulfate (CAS 151-21-3) were bubbled out with high purity nitrogen.

All measurements were carried out using a buffer solution of Britton–Robinson (BR) 0.1 mol L^−1^ prepared by mixing specific volumes of acids (phosphoric acid-H_3_PO_4_, boric acid-H_3_BO_3_, and acetic acid-CH_3_COOH) and adjusted with concentrated NaOH.

### 2.2. Instrumentation

Electrochemical measurements were conducted by the WaveNow potentiostat/galvanostat, controlled by AfterMath software 1.2.5968 (PINE research, Durham, NC, USA) employing a three-electrode cell consisting of a saturated Ag/AgCl reference electrode MF-2056 (BASi, West Lafayette, IN, USA), a graphite bar as the auxiliary electrode, and a working electrode based on a carbon paste electrode with MWCNT (MWCNT-CPE). The pH measurements were performed on a 450 pH/ion meter (Cole-Parmer, Corning, NY, USA). Additionally, an analytical model CPA224S balance was used (Sartorious, Goettingen, Germany). The scanning electron microscopy images were acquired by the microscope JSM-IT300 (InTouchScop, Tokyo, Japan) for carrying out the MWCNT-CPE sensor surface characterization. The studies of electrochemical impedance spectroscopy were performed using a potentiostat Autolab PGSTAT302N with FRA32M module (Metrohm, Utrecht, The Netherlands).

### 2.3. Working Electrode Preparation

**Figure 2:**
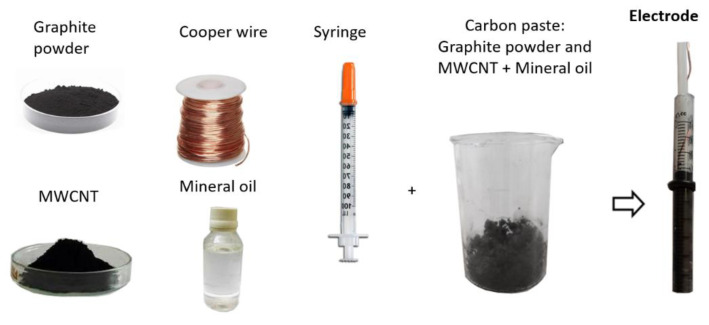
Materials and preparation of the working electrode.

The modified working electrode was prepared using a graphite powder and mineral oil mix in a 3:2 ratio. This mixture also contains a particular portion of MWCNT. Different percentages were tested, 0, 5, 7.5, 10, and 15%, to evaluate the optimal content of MWCNT. The final blend was collocated inside a 1 mL syringe tube (30 × 6 mm) and compacted with the syringe’s plunger, placing it on a flat surface to eliminate excess air. Finally, a copper wire acting as electrical contact was inserted at one end of the syringe. Figure 2 shows the composition of the working electrode.

### 2.4. Sample Preparation

Paracetamol, diclofenac, naproxen, aspirin, ascorbic acid, glucose, and sodium dodecyl sulfate stock solutions were prepared from analytical-reagent-grade chemicals in the BR buffer solution. The first stock of solutions was prepared at a fixed concentration of 0.5 mmol L^−1^ for each drug in order to use them in the optimization stage of the sensor. The second stock containing 81 samples was prepared according to a 3^4^ factorial design (FD) [42], in the range of 0.5 and 80 µmol L^−1^ considering fixed concentrations of interferents (ascorbic acid at 80 µmol L^−1^, glucose at 18.5 µmol L^−1^, and sodium dodecyl sulfate at 22 µmol L^−1^). This concentration range was chosen considering the reported ranges in the literature for the different NSAID voltammetric determination in different matrices [7,14,15,16,17]. Finally, the concentrations of the excipients were calculated based on the content present in a commercial drug tablet.

The concentrations in each mixture sample were selected to generate a simplified model space without trends and drifts; this ensures appropriate data modeling out of being conditioned by a previous data sample measured. Additionally, an extra set was formed by 10 synthetic solutions prepared in the same way but with concentrations generated randomly inside the model space described.

### 2.5. Electrochemical Analysis and Procedure

The analysis of the NSAIDs was firstly conducted by CV using the supporting electrolyte (BR buffer at pH 7) and fixed concentrations of 0.5 mmol L^−1^ for each drug (paracetamol, diclofenac, naproxen, and aspirin) under analysis. As a working electrode, the different MWCNT-CPEs containing individual percentages of MWCNT (as mentioned in Section 2.3) were used. The potential range was from 0 to 1.3 V at a scan rate of 0.1 V s^−1^. In this way, it is possible to study nanotubes’ optimal content in the sensor’s structure and execute a pH review with the NSAIDs-BR system to choose the maximum anodic current intensity for the analytical quantification drugs.

Meanwhile, the simultaneous quantification of the drugs was carried out through DPV. A second-order model regression based on a Box–Behnken Design (BBD) was applied to improve the technique’s parameters [43]. The BBD considered three levels and was used to optimize the four variables related to the DPV technique, searching to maximize the anodic current peak and sensitivity. The DPV parameters of interest were the step potential (V), interval time (s), modulation time (s), and modulation amplitude (V). Finally, the maximum anodic current intensity was predicted using polynomial regression.

### 2.6. Data Processing

**Figure 3:**
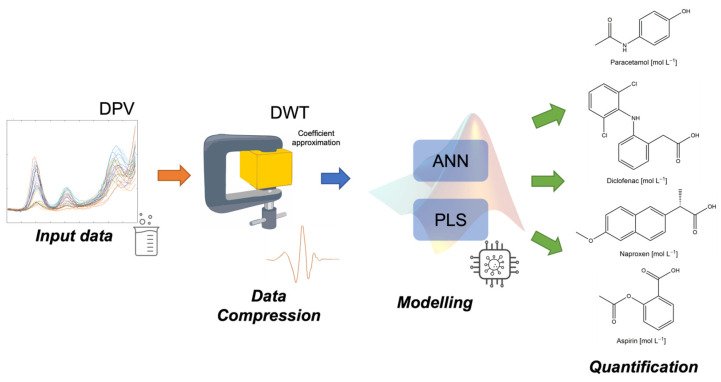
Scheme for modeling DPV data for the multi-quantification of the four NSAIDs studied.

The data processing for multi-analyte quantification by PLS and ANN models requires improved voltammograms to overcome the signals’ extreme complexity and high dimensions (see Figure 3). The measured voltammetric data were represented by a matrix of dimension [177 × 81] (intensities × number of samples). The DWT technique was used as a pretreatment tool, using the third-level wavelet decomposition of Daubechies function (db4) to compress DPV voltammetric records. In this way, only approximation coefficients were selected considering the similarities between the original voltammograms and those recovered [44,45], achieving a mean comparison factor of 0.9823. Thus, the final data matrix dimension to feed the calibration models was [28 × 81] (approximation coefficients × number of samples).

The PLS modeling/reduction data were integrated for entry values, regression coefficients and the number of components. For the prediction process of the PLS model (data relation), the previously compacted DPV records (X-experimental, [28 × 81] matrix) and the concentrations of the four drugs (Y-prediction, [4 × 81] matrix) were used. This process generated regression coefficients that are the principal values to find out the relation between the matrix data [28 × 81] and drug concentrations [4 × 81]. Added to this, the concentrations of the NSAIDs were used to find a pattern based on the correlation of the DPV spectral data. One important characteristic of the regression model is the performance related to the number of components. A different number of components was tested to achieve the best performance and correlation. Few components result in a model with a poor correlation and too many components result in a model sensitive to noise [35,36].

Meanwhile, the ANN calibration model was based on an MLP. The number of approximation wavelet coefficients established the input layer of the MLP. In contrast, the output layer was defined using one neuron for each analyte (paracetamol, diclofenac, naproxen, and aspirin) to be quantified since it is associated with the matrix of concentrations of [4 × 81]. The hidden layers were established through a trial-and-error process, modifying the number of neurons in the layers until an appropriate number of neurons was found to obtain a satisfactory linear regression coefficient. The final MLP model contained a structure of 28 × 16 × 8 × 4 neurons (28 input, 16 for the first hidden layer, 8 for the second hidden layer, and 4 for the output).

A Box–Behnken design was executed using Minitab^®^ Statistical Software version 18 (Minitab LLC, State College, PA, USA). On the other hand, different processing and modeling stages were performed in MATLAB^®^ R2021a (MathWorks, Natick, MA, USA) using Wavelet, Statistics and Machine Learning, and Deep Learning toolboxes, together with specific routines programmed by the authors.

## 3. Results and Discussion

### 3.1. System Characterization

**Figure 4:**
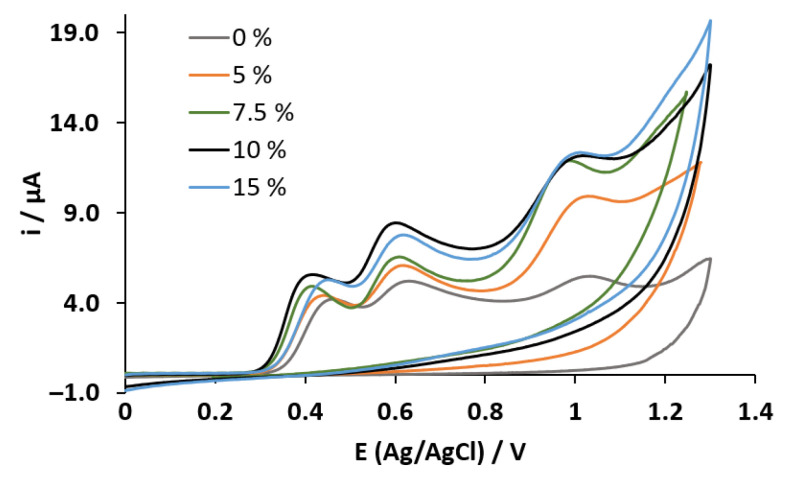
CVs obtained using different MWCNT percentages in the working electrode for a sample containing a mixture of paracetamol, diclofenac, naproxen, and aspirin.

The CV was performed for preliminary electrochemical studies of the NSAIDs, using a potential window from 0 to 1.3 V and the MWCNT-CPE working electrodes containing different percentages of carbon nanotubes in their composition. Figure 4 shows the obtained voltammograms for a mixture of the four drugs at fixed concentrations of 0.5 mmol L^−1^ for each drug using the different percentages of MWCNT in the electrode’s composite mixture. In the first scan (anodic direction), the oxidation peaks corresponding to paracetamol (0.446 V) and diclofenac (0.629 V) are visible. In contrast, naproxen and aspirin are not evident at typically reported oxidation potentials due to overlapped peak effect (observed around 1.05 V), regardless of the nanotube percentage in the working electrode. Contrary, when reversing the sweep (cathodic direction), no reduction peaks were observed, this behavior is typical of an electrochemical–chemical process. It is well known that NSAID oxidation products suffer a subsequent chemical process with final products no longer electroactive. Based on this study, the highest anodic peak current for paracetamol was achieved using 10% of MWCNT in the electrode’s paste, so this proportion was selected for further experiments.

**Figure 5:**
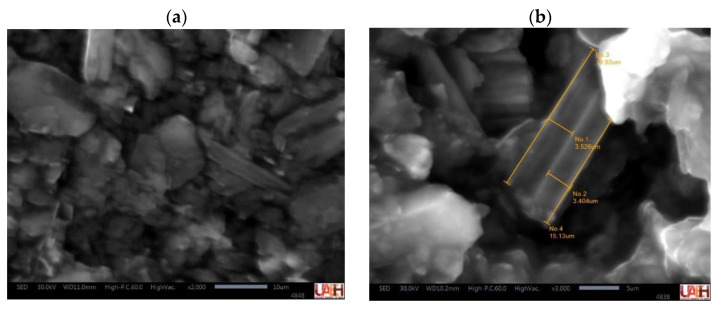
Scanning electron microscopy characterization. (**a**) CPE surface structure. (**b**) MWCNT-CPE surface with carbon structure identification. The images were acquired by applying a primary beam operating voltage of 30 kV, and a magnification ratio of 1:2000 for both samples.

**Figure 6:**
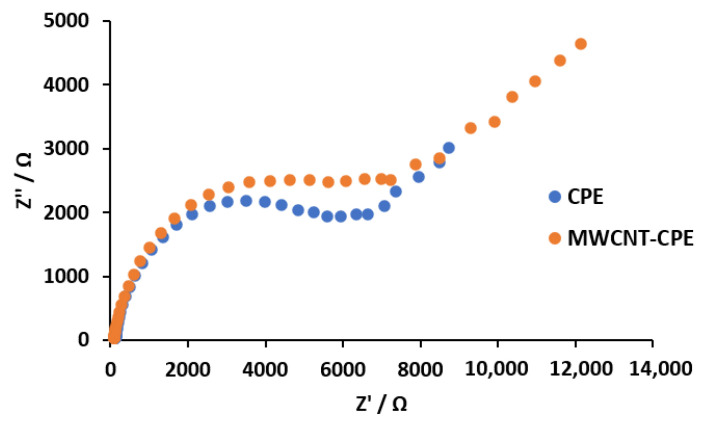
Impedance spectra (Nyquist plots) for the CPE and MWCNT-CPE at the open circuit potential, in a solution containing 1.0 mmol L^−1^ K_3_[Fe(CN)_6_]/K_4_[Fe(CN)_6_].

The presence of the carbon nanotubes on the MWCNT-CPE sensor was confirmed by SEM and EIS (Figure 5 and Figure 6, respectively), observing the surface morphology composition and the electron transfer kinetics behavior of the proposed electrode (nanotubes) and the conventional CPE sensor. A detailed description of these processes is as follows.

The images for both sensors were acquired at 30 kV and a magnification ratio of 1:2000. In Figure 5a, graphite powder adhered to the mineral oil (agglomeration) is observed on the surface of the CPE electrode; this structure allows having a semi-solid electrode with electrical conduction [46]. Moreover, Figure 5b shows the MWCNT-CPE surface where the agglomeration of uniform MWCNTs coated with the binder (0.5–1.0 µm thickness of the mineral oil layer [46]) with a size of 3.3 ± 0.5 µm [47]. Under these conditions, it was possible to visualize tubular MWCNTs [48] adhered to the mineral oil.

The EIS analysis was performed in an open circuit potential and a solution at 1.0 mmol L^−1^ of K_3_[Fe(CN)_6_]/K_4_[Fe(CN)_6_]. The analysis of the Nyquist plot presented in Figure 6 shows a slight increase in the electrical resistance of the modified electrode MWCNT-CPE compared to the electrode CPE. The presence of the MWCNTs at the structure of the modified electrode resulted in a charge transfer resistance (Rct) of 234.45 Ω in comparison to the MWCNT-CPE of Rct = 248.75 Ω. This result is in accordance with Figure 4, where better electrocatalytic performance is observed as all anodic peaks move to lower potentials and an increase in the capacitive current is also observed [49,50,51,52]. In this sense, the presence of MWCNT increases the electrode’s surface area in contact with the sample, enhancing the measured analytical signal. Thus, the redox reactions of the analyte are more efficient at the MWCNT-CPE surface.

**Figure 7:**
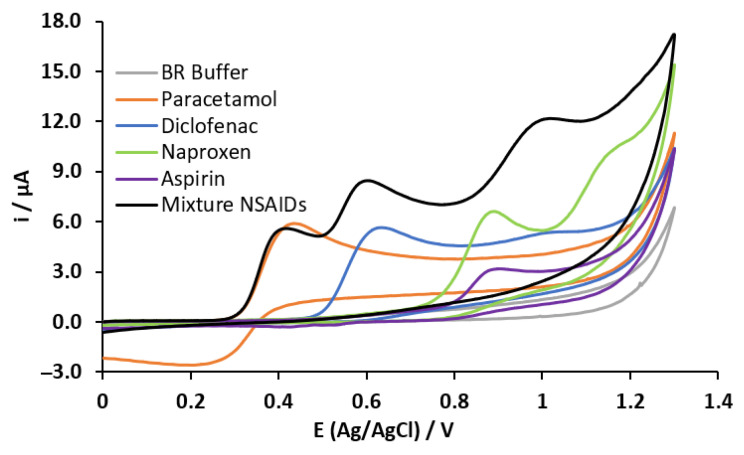
CVs for each NSAID under study: a mixture containing the four NSAIDs and the supporting electrolyte (BR).

Once the optimized MWCNT-CPE sensor was established, the next step was the execution of the new CV experiment, to identify particularities in the voltammograms related to the drugs under study, being applied in a solution of 0.5 mmol L^−1^ of the drug standards in BR buffer at pH 7. The experimental stage was performed using a mixture at fixed concentrations of each of the four NSAIDs and BR Buffer (blank). In this manner, it is possible to observe, from the sweep (in the anodic direction), characteristic oxidation peaks located at the potentials of 0.446 and 0.880 V that are mainly related to paracetamol and aspirin, respectively. On the other hand, two representative oxidation peaks were observed at potentials of 0.624 and 1.02 V for diclofenac, as well as 0.888 and 1.12 V for naproxen. Finally, only one reduction peak was observed at 0.263 V in the cathodic direction, corresponding to paracetamol (see Figure 7).

Additionally, in Figure 7, it is possible to observe the voltammogram obtained for the mixture of the four NSAIDs. Here, the oxidation peak of paracetamol and diclofenac can be observed in the mix at the mentioned potentials. Nevertheless, this effect does not happen for aspirin and naproxen because there is a strong overlap of oxidation peaks between both drugs. Additionally, it is possible to identify a signal contribution from diclofenac due to its second oxidation peak. Finally, it is important to note an absence of the cathodic peak in the CV; this indicates additional chemical reactions of the oxidation products of paracetamol with the diclofenac and/or the naproxen. The experiment’s purpose is to observe the independent behavior of each drug as well as their mixture since the quantification of the drugs was performed simultaneously.

Considering the CV anodic response related to the oxidation of the NSAID mixture, a pH study evaluating values from 7 to 10 was also carried out. Table 1 reports the current intensities obtained in the experiment; additionally, it is possible to observe that the anode currents increase with a pH of 10 for the mix of drugs. Other pH values such as 6 and 11 were not considered because the mixture of drugs was insoluble in the BR buffer of 0.1 mol L^−1^ [49].

For the multi-analyte quantification stage, a DPV technique was used, as mentioned in Section 2.5. Twenty-seven runs were performed using the same mixed sample of NSAIDs (BR buffer at pH 10) to optimize DPV parameters. The NSAID mixture voltammograms were measured using the selected MWCNT-CPE sensor and changing the four parameters involved in the DPV technique every time. The following Equations (1)–(3) describe the second-order regression model for modeling the drug’s maximum anode current intensity. Here, *Y*_1_ is related to paracetamol, *Y*_2_ to diclofenac, and *Y*_3_ to naproxen and aspirin together.

(1) $$Y_{1}=1.747+0.22X_{1}+0.352X_{2}-0.935X_{3}+1.082X_{4}\;-0.476X_{1}^{2}-0.431X_{2}^{2}-0.308X_{3}^{2}-0.012X_{4}^{2}\;-0.309X_{1}X_{2}+0.211X_{1}X_{3}+0.209X_{1}X_{4}+0.297X_{2}X_{3}+0.66X_{2}X_{4}+1.507X_{3}X_{4}$$

(2) $$Y_{2}=1.060+0.069X_{1}+0.278X_{2}-0.539X_{3}+0.613X_{4}\;-0.393X_{1}^{2}-0.353X_{2}^{2}-0.153X_{3}^{2}+0.010X_{4}^{2}\;+0.105X_{1}X_{2}+0.123X_{1}X_{3}+0.156X_{1}X_{4}+0.223X_{2}X_{3}+0.373X_{2}X_{4}-0.885X_{3}X_{4}$$

(3) $$Y_{3}=0.626+0.204X_{1}+0.130X_{2}-0.318X_{3}+0.490X_{4}\;-0.014X_{1}^{2}-0.125X_{2}^{2}-0.007X_{3}^{2}-0.055X_{4}^{2}\;-0.144X_{1}X_{2}+0.201X_{1}X_{3}+0.296X_{1}X_{4}+0.242X_{2}X_{3}+0.377X_{2}X_{4}-0.554X_{3}X_{4}$$

Minitab^®^ V.18 was used to find the best settings for each response based on the attained DPV parameters. The best definition of each NSAID response was evaluated to achieve the maximum anodic current peak. The optimal parameters obtained from the BBD’s second-order model regression were a step potential of 0.00585 V, an interval time of 0.75 s, a modulation time of 0.05 s, and a modulation amplitude of 0.05 V for the mixture of the four NSAIDs.

**Figure 8:**
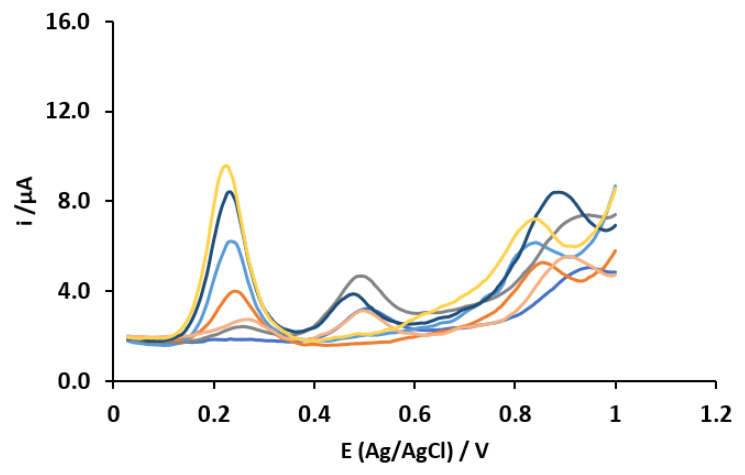
DPVs of some of the 81 processed samples at different concentrations of the NSAIDs.

Using these optimal parameters for the DPV (mentioned above), the 81 prepared samples following the FD were measured using a potential sweep from 0 to 1.05 V. During this procedure, the current is measured in a quiescent solution without any oxygen removal from the sample and surface regeneration of the MWCNT-CPE sensor. Each obtained voltammogram consists of 177 current values at different concentrations of the NSAIDs in a range of 0.5 to 80 µmol L^−1^. Figure 8 shows the voltammograms of this analysis.

This last procedure was also employed to measure the set of 10 random samples used to validate the computer models programmed for the simultaneous quantification of the NSAIDs.

### 3.2. Quantification of NSAIDs by Chemometric Approach

In the first approach, PLS modeling was conducted considering for the training stage the matrix of independent variables (X, experimental) defined by the set of pretreated voltammograms that form a matrix of dimensions [28 × 81]. The matrix of prediction values (Y, estimation) with dimensions [4 × 81] is defined by the number of NSAIDs analyzed and the total samples. In the same way, for the testing stage, the X data of ten synthetic solutions [28 × 10] and Y data of [4 × 10] were used. An iterative process using cross-validation was performed to establish an adequate number of components considering the performance of the linear correlation between expected and obtained NSAID concentration values on each try to avoid overfitting. This analysis showed that choosing twelve principal components was enough to achieve the minimal error between obtained and expected NSAID concentration values.

Subsequently, a comparison analysis between the obtained vs. expected concentrations was performed for the training and testing subsets to determine the PLS performance in the simultaneous quantification of drugs. The linear regression of the comparison was a measure of the goodness of the model; given in ideal conditions, it should yield the diagonal identity line (slope 1 and intercept 0). Table 2 summarizes this information reporting the main parameter values of the regression analysis, showing that in the case of naproxen and aspirin (R values of 0.551 and 0.538, respectively), the PLS was unable to perform the determination of testing concentrations properly. This shows the need to use non-linear modeling that allows for the suitable quantification of these drugs.

On the other hand, for the modeling based on ANN, the same training and testing matrices (of dimensions [28 × 81] and [28 × 10], respectively) used in the construction of the PLS were used. Nevertheless, all data sets were normalized between [−1,1] interval to simplify operations and lower the computational cost. After testing different combinations of the activation functions, the best results were obtained using: (1) *purelin* for the input layer, (2) *tansing* for the two hidden layers, and (3) *purelin* for the output layer. The training algorithm selected was Bayesian regularization; this algorithm considers the global error and the value of the weight of every single connection used in MLP [39]. The parameters used for learning conditions were a training error of 0.015, a learning rate of 0.05, and a momentum of 0.5.

**Figure 9:**
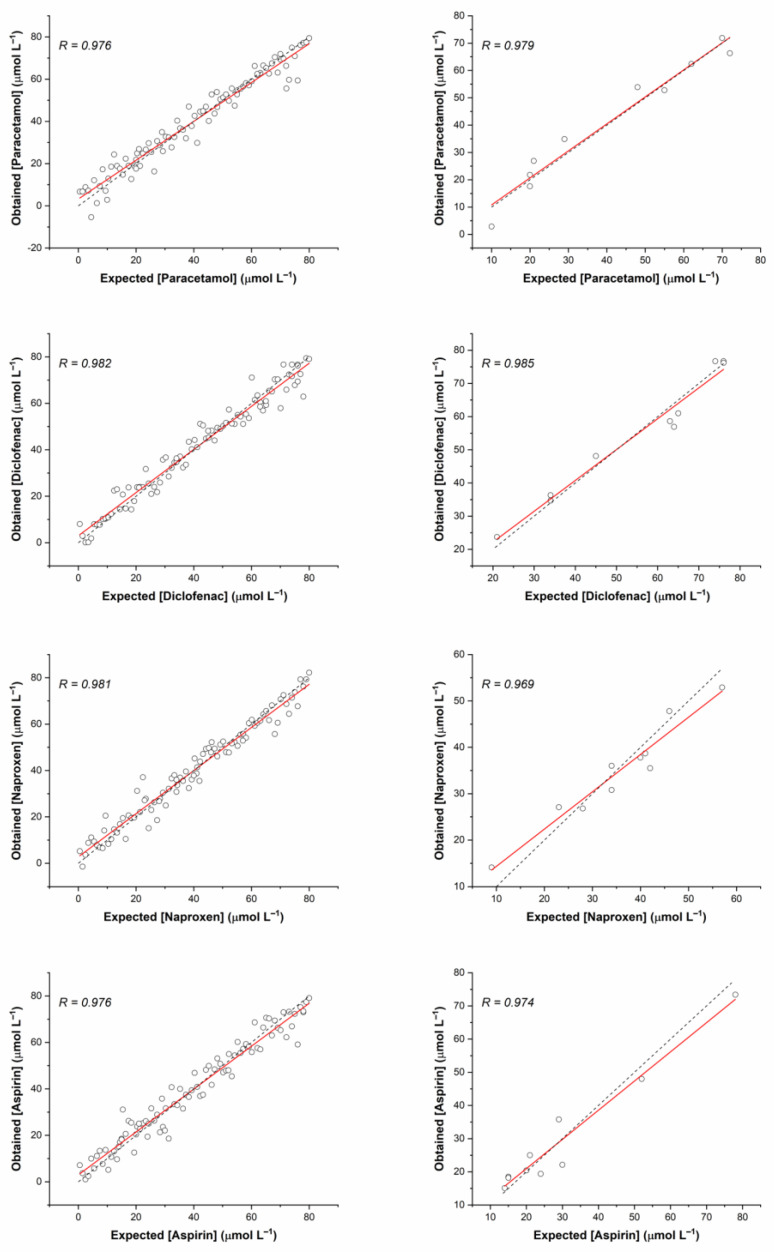
Obtained vs. expected concentration of NSAIDs using MLP. The solid line in each graph corresponds to the regression of data (correlation coefficient R). The plots on the left indicated the training set and on the right the testing set.

The trained MLP was used to determine the performance in the simultaneous quantification task of NSAIDs and evaluated the corresponding relationships between the concentrations obtained and those expected for the training or testing stages. Again, the linear regression obtained from the comparison was a measure of the model’s goodness. The high level of linearity allows the linear regression coefficients of the test data obtained to be very close to one (R ≥ 0.969). Similarly, the limit of detection (LOD) and the limit of quantification (LOQ) were evaluated according to the MLP’s testing response; their calculation was as follows: LOD = 3 *σ/m* and LOQ = 10 *σ/m*, where *m* is the slope of the calibration curve and *σ* is the standard deviation of the response (see Table 3). Table 4 reports the main parameter values of the regression analysis. In this case, the ideal situation is fulfilled in all cases (at the 95% confidence level). The comparative graphs between the real concentrations of paracetamol, diclofenac, naproxen, and aspirin and those predicted with the adjusted MLP model are shown in Figure 9. These values allow us to deduce that there is a high level of adjustment accomplished by the MLP model that permits the interpretation of the synthetic test data.

Additionally, to compare the preciseness of the prediction, the recovery yield (*Ry*) for each PLS and ANN model [53] was calculated. *Ry* is defined by Equation (4):

(4) $$Ry=\frac{\sum_{i=1}^{N}100\cdot \left(1+\frac{y_{i}-y_{exp,i}}{y_{exp,i}}\right)}{N}$$

where $y_{i}$ is the *i*-th obtained concentration value, $y_{exp,i}$ is the *i*-th expected concentration value, and *N* is the size of the external test set.

The average *Ry* values for the PLS and ANN models (training and testing) are shown in Table 5.

The *Ry* values showed a perspective of the variability or fluctuation of the information contained (DPV data) that supports the prediction of drug concentrations from random selection data.

The givens obtained by the determination of the *Ry* values for the four drugs on the ANN model predict the nearby concentration data (109.9% average), indicating sufficient analytical information. On the other hand, the four systems for the PLS model showed values out of the accuracy (>20%) on the prediction information. This support the information (PLS analysis) on the differences in the random data that cannot be reduced by the model limitations for the concentration quantification.

## 4. Conclusions

The present work reported a potential tool for the determination and voltammetric analysis of the main NSAIDs used in clinical practice through the successful combination of the DPV and DWT-ANN techniques. The DPV showed to be adequate in studying these drugs, allowing us to obtain records of mixtures of paracetamol, diclofenac, naproxen, and aspirin quickly and reproducibly. Meanwhile, the chemometric combination of DWT-ANN was the most appropriate option to achieve the simultaneous quantification of the drugs because it allows the interpretation of the complexity of the voltammograms due to the overlapping of peaks. On the other hand, the quantification models based on DWT-PLS showed a lower performance with the test data than those obtained with ANN. This fact indicates that linear modeling is not appropriate for this application.

The incorporation of MWCNT into the composite mixture of the working electrode and optimizing the DPV parameters through a Box–Behnken design allowed the improvement of the analytical signal with a higher sensitivity of the sensor in the determination of drugs at low concentration levels. The integration of MWCNT demonstrated good electrical conductivity and allowed a better transference of the electrons in the electrode’s surface. This result was corroborated by the presence of carbon nanotubes (fusion) on the surface of the working electrode through the SEM and electron transfer (conductivity) by the analysis of the EIS techniques.

Therefore, this work represents a low-cost and short-time methodology for NSAID analysis, demonstrating the advantages of the use of MWCNT-CPE along with chemometric tools as part of a promising methodology with encouraging on-field clinical analysis, the examination of pharmaceutical samples containing these drugs at high concentrations or more complex samples such as hospital wastewater, urine, and blood serum, where the concentration of the NSAIDs is at the trace level. The proposed methodology could be used as a supporting technique (high sensitivity and low detection limits), a comparative data technique (simultaneous detection), or a potential substitute for traditional time-consuming methods such as HPLC.

## Figures and Tables

**Table 1:** Study of current intensities at different pH for a mixture of drugs.

pH	Maximum Peak Current Intensity of Drugs (µA)		
	Paracetamol	Diclofenac	Naproxen/Aspirin
7	4.84	7.62	11.96
8	5.12	7.88	12.11
9	6.12	8.46	17.70
10	7.26	8.81	18.10

**Table 2:** Linear regression parameters for the comparison of the obtained vs. expected data with the training and testing data sets using a PLS model with 12 components.

NSAID	Training					Testing				
	R	m	Δm	b	Δb	R	m	Δm	b	Δb
Paracetamol	0.827	0.684	0.196	1.274 × 10^−5^	9.098 × 10^−6^	0.974	0.865	0.328	8.771 × 10^−6^	1.520 × 10^−5^
Diclofenac	0.778	0.605	0.206	1.654 × 10^−6^	9.869 × 10^−6^	0.947	0.964	0.532	−3.590 × 10^−5^	3.107 × 10^−5^
Naproxen	0.801	0.641	0.202	1.426 × 10^−5^	9.211 × 10^−6^	0.551	0.327	0.836	2.085 × 10^−5^	2.968 × 10^−5^
Aspirin	0.741	0.549	0.210	1.762 × 10^−5^	9.515 × 10^−6^	0.538	0.327	0.836	2.085 × 10^−5^	2.968 × 10^−5^

**Table 3:** LOD and LOQ evaluated the MWCNT-CPE for the testing stage using an MLP architecture with two hidden layers.

NSAID	LOD (µmol L^−1^)	LOQ (µmol L^−1^)
Paracetamol	15.4	51.3
Diclofenac	10.6	35.4
Naproxen	8.89	29.5
Aspirin	13.4	44.8

**Table 4:** Linear regression parameters for the comparison of the obtained vs. expected data with the training and testing data sets using an MLP architecture with two hidden layers.

NSAID	Training					Testing				
	R	m	Δm	b	Δb	R	m	Δm	b	Δb
Paracetamol	0.976	0.919	0.044	3.238 × 10^−6^	2.025 × 10^−6^	0.979	0.989	0.166	8.897 × 10^−7^	7.692 × 10^−6^
Diclofenac	0.982	0.929	0.038	2.987 × 10^−6^	1.820 × 10^−6^	0.985	0.930	0.134	3.569 × 10^−6^	7.801 × 10^−6^
Naproxen	0.981	0.930	0.039	2.762 × 10^−6^	1.765 × 10^−6^	0.969	0.804	0.166	6.299 × 10^−6^	6.255 × 10^−6^
Aspirin	0.976	0.924	0.043	2.975 × 10^−6^	1.957 × 10^−6^	0.974	0.879	0.165	3.398 × 10^−6^	5.870 × 10^−6^

**Table 5:** *Ry* values of the variability of information for the PLS and ANN models.

NSAID	*Ry* (%)			
	PLS		ANN	
	Training	Testing	Training	Testing
Paracetamol	174.5	171.2	122.5	114.9
Diclofenac	102.6	119.8	97.8	103.7
Naproxen	149.5	62.7	118.4	119.9
Aspirin	96.2	71.3	100.9	103.9

## Data Availability

The data that support the findings of this study are not publicly available. However, data can be requested from the authors and fulfilling the authorization requirements of the involved universities in this research.
